# User considerations in assessing pharmacogenomic tests and their clinical support tools

**DOI:** 10.1038/s41525-018-0065-4

**Published:** 2018-09-11

**Authors:** Gouri Mukerjee, Andrea Huston, Boyko Kabakchiev, Micheline Piquette-Miller, Ron van Schaik, Ruslan Dorfman

**Affiliations:** 1 GeneYouIn Inc., 156 Front St. W., Toronto, ON Canada; 2 Lunenfeld-Tanenbaum Research Institute, Mount Sinai Hospital, Toronto, ON Canada; 3 Leslie Dan Faculty of Pharmacy, University of Toronto, Toronto, ON Canada; 4 International Expert Center Pharmacogenetics, Department of Clinical Chemistry, Erasmus University Medical Center, Rotterdam, The Netherlands

## Abstract

Pharmacogenomic (PGx) testing is gaining recognition from physicians, pharmacists and patients as a tool for evidence-based medication management. However, seemingly similar PGx testing panels (and PGx-based decision support tools) can diverge in their technological specifications, as well as the genetic factors that determine test specificity and sensitivity, and hence offer different values for users. Reluctance to embrace PGx testing is often the result of unfamiliarity with PGx technology, a lack of knowledge about the availability of curated guidelines/evidence for drug dosing recommendations, and an absence of wide-spread institutional implementation efforts and educational support. Demystifying an often confusing and variable PGx marketplace can lead to greater acceptance of PGx as a standard-of-care practice that improves drug outcomes and provides a lifetime value for patients. Here, we highlight the key underlying factors of a PGx test that should be considered, and discuss the current progress of PGx implementation.

## Introduction

Pharmacogenomics (PGx) is the study of inherited genetic information that influences drug response and determines drug behavior. This inherently personalized approach to medicine can improve drug efficacy and has the potential to engage patients in their own health care, leading to better treatment adherence and outcomes. Implementation projects using PGx-guidance for drug therapy have demonstrated a high frequency of pharmacogenetically relevant genomic variants in the general population and a potential value of PGx-guided drug selection.^1,2^ Research programs in PGx implementation are underway in Canada (Go-PGx, IMPACT), EU (U-PGx), and US (eMERGE) and publications reviewing clinical applications of PGx have summarized challenges and provided recommendations for moving forward.^3,4^

The use of PGx as a tool for evidence-based medication management is gaining acceptance beyond academic medical centers and hospital systems with many users—individuals, health professionals, and health plans—expressing an interest in using PGx tests to predict efficacy and side effects of drugs. In addition to PGx tests used in medical centers and hospital settings, there are numerous commercially available PGx-based decision support tools (DSTs) on the market utilizing different genetic panels (Supplementary Table 1) with dissimilar medication coverage. Bousman and Dunlop examined the degree of agreement in medication recommendations between four commercial PGx-based DSTs with published data in context of major depressive disorder (MDD).^5^ Despite concordance in many results, a level of disagreement in medication recommendation was observed for antidepressants, anxiolytics/hypnotics, and antipsychotics. Therefore, tests cannot be assumed to be equivalent or interchangeable and users need to evaluate available tests before making a choice. Due to limited familiarity with the technology, users often face a challenge in comparative evaluation of available tests. Here, we explain the different features of a PGx service, such that users can make informed decisions to identify a test that fits their needs.

## Pharmacogenomic tests

Two approaches to PGx testing have been adopted: reactive testing and pre-emptive testing. In reactive testing, genetic tests are ordered on an ‘as needed’ basis. If the results are deemed to be of interest: the patient is likely to need, or has side effects from a high-risk drug with PGx recommendations. In pre-emptive testing, drug response genes are tested in anticipation of future prescription events, providing a lifetime value for the test. Results for high-risk drugs with PGx recommendations can be made available prior to prescribing decision, consistent with the vision that in every prescribing decision, an individual’s genomic variation will be considered an inherent patient characteristic as are age, weight, renal function, and allergy status.^4^

### Pharmacogenes impact drug response

Drug response is highly variable and some of this variation is due to inherited genetic variants. Genetic variants are estimated to affect between 20–95% of response variability depending on the drug.^6^ Variants influencing drug response are predominantly located in genes encoding drug-metabolizing enzymes and transporters (the ADME genes), drug targets, or human leukocyte antigen alleles. Variations can occur in regulatory regions of the gene affecting level of expression, as well as in the coding region affecting the function of the gene, causing high or low exposure to the drug, increased formation of toxic metabolites, increased/decreased interactions with drug targets, or activation of the immune system leading to idiosyncratic drug toxicity.

There are many examples of PGx variants impacting drug efficacy and safety.^7^ Ultrarapid metabolizers of CYP2D6 can suffer from life threatening respiratory depression when prescribed codeine; poor metabolizers of CYP2C19 have impaired ability to activate clopidogrel, reducing the medications’ therapeutic efficacy, and HLA variants help prescribers prevent hypersensitivity reactions to abacavir, carbamazepine, and allopurinol. Clinical trials, regarded as the gold-standard for assessing clinical utility, have been conducted for PGx variants associated with warfarin,^8–10^ abacavir,^11^ statins,^12^ and clopidogrel.^13^ Validation studies are needed to assess clinical impact and utility of other drug–gene interactions reported.

### Curated databases with PGx recommendations

Curated databases, such as the Clinical Pharmacogenetics Implementation Consortium (CPIC), the Dutch Pharmacogenetics Working Group (DPWG), the Pharmacogenomics Knowledge Base (PharmGKB), and the Canadian Pharmacogenomics Network for Drug Safety (CPNDS) are important sources of information on PGx variants that affect drug response and prescribing guidelines associated with these variants. Both CPIC and DPWG provide therapeutic recommendations for more than 40 well-known gene-drug pairs,^14^ while the CPNDS clinical recommendation group has published dosing guidelines for adult and pediatric patients for a few select drugs.

### PGx assays impact sensitivity and specificity of tests

Various technologies assess pharmacogenes and selection of the appropriate assay depends on the target population, prior characterization of genetic variants, automation requirements, and cost.

Targeted genotyping assays probe for preselected variants with well-defined drug-gene interactions. 'Ready-made' commercial genotyping assays are often multiplexed PCR-based technologies using TaqMan® hydrolysis probe chemistry (QuantStudio) or Illumina VeraCode® ADME Core Panel. Other examples include: bead-based immunoassay testing (Luminex), microarrays (Affymetrix), and MassArrays (Sequenom iPLEX® ADME Pharmacogenetic Panel).

Targeted genotyping assays offer robust interpretation well suited for automation of PGx reporting. The assays typically include well-studied pharmacokinetic and pharmacodynamic markers of the drug metabolizing enzymes (i.e., *CYP2D6*, *CYP2C19*, *CYP3A5*, *DPYD*, *TPMT*, and *UGT1A1*), selected drug transporters (*SLCO1B1*), receptors (*VKORC1*), and other genes associated with drug response.

**Fig. 1:**
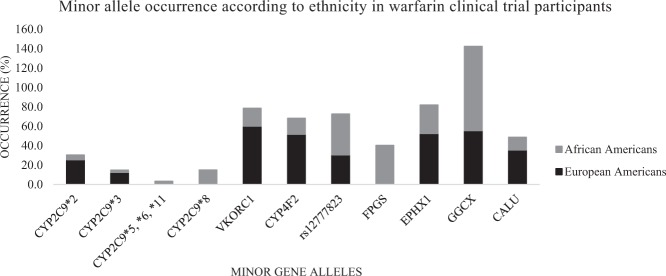
Occurrence of minor alleles as per ethnicity in the University of Alabama warfarin clinical trial shown by percentage of participants possessing minor alleles^10^

One caveat of targeted genotyping is that some assays may include only variants common in specific ethnic populations (usually Caucasian), thus missing other ethnicity-specific alleles. Based on the population under investigation, the sensitivity and specificity of a PGx genotyping assay can vary, depending on the genetic variants included. For example, two reduced function variant alleles important for response to the anticoagulant warfarin (CYP2C9*2 and CYP2C9*3), are common in Caucasians but not African-Americans. Lack of inclusion of reduced function alleles common to African-Americans (CYP2C9*5, *6, *8, *11) undermined early warfarin PGx clinical trials.^8^ Subsequent warfarin trials in the US accounted for ethnicity-specific *CYP2C9* and *CYP4F2* alleles, improving prediction of warfarin maintenance dose^10^ (Fig. 1).

PGx sequencing assays utilizing whole-exome sequencing (WES) and whole-genome sequencing (WGS) technologies are variant agnostic and all genetic variants are identified. Nevertheless, sequencing has not been widely implemented due to: (i) higher cost, (ii) the absence of functional characterization for many less common variants, and (iii) challenges in the resolution of copy number variations and long repeat elements. For example, exome sequencing data for 12 pharmacogenes of the cytochrome gene family identified multiple rare variations of potential significance, however, most lacked functional characterization, posing a challenge on how to act on these findings.^15^ Also, it should be recognized that WES may not cover some variants with well-defined PGx recommendations (e.g., rs9934438 of *VKORC1*).

For pharmacogenes such as *G6PD*, sequencing technologies offer the most effective solution. G6PD deficiency affects an estimated 4.9% of the world’s population. Hundreds of genetic variants of *G6PD* have been described, with wide ranging effects on enzyme activity. Designing a genotyping assay encompassing all functional *G6PD* gene variants is challenging and sequencing stands as the most sensitive solution.

Several PGx panels with a common core of pharmacokinetic variants in *CYP2C19* and *CYP2D6*, as well as unique combinations of other variants have been developed to guide prescription of antidepressants, antipsychotics, and antianxiety drugs. Randomized clinical trials utilizing these psychiatric PGx tests show PGx-guided therapy improved tolerability and safety of treatment.^16–18^ Of note, psychiatric PGx tests include different combinations of variants (Supplementary Table 1), each with a limited effect size.

A study examining the degree of agreement in medication recommendations between four commercial PGx-based DSTs for patients with MDD observed a level of disagreement in recommendations.^5^ Agreement was highest for mood stabilizers (84%), while it was 55–56% for antidepressants, anxiolytics/hypnotics, and antipsychotics. The authors were unable to determine, in most cases, the cause of inter-DST disagreements on recommendations due to the unknown quality of the evidence base used to formulate the proprietary algorithms, but hypothesized the disagreement was a result of differences in the genes/variants tested, phenotyping strategies, and algorithms used to predict drug–gene interactions. A systematic literature review examining *CYP2D6*, *CYP2C19*, *CYP2C9*, *CYP1A2*, *CYP3A4*, *HTR2C*, *HTR2A*, and *SLC6A4* found the strongest gene-outcome associations for psychiatric pharmacotherapy with *CYP2D6* and *CYP2C19*,^19^ which was endorsed by a recent study examining treatment outcome in psychiatric care with only *CYP2D6* and *CYP2C19* genetic information.^20^ Variants with individually small effects may be combined into a sufficiently predictive test to guide drug treatment. However, large-scale studies are needed to replicate/validate these tests and model the combinatorial effects of multiple variants.^21^

A bias towards including ‘historic’ variants appears in many psychiatric PGx tests. Meta-analysis studies demonstrated an association between depression and response to antidepressants and the long/short forms (known as L/S alleles) of promoter repeats in the *SLC6A4* gene (also known as 5-HTTLPR, 5-HTT, or serotonin transporter, SERT).^22,23^ Studies have identified missense variants in the coding region of *SLC6A4* and intronic SNPs that affect response to SSRIs and SSRI-induced side effects.^24,25^ However, the relative effect size of each variant remains unknown. Consequently, most psychiatric PGx tests focus on the promoter variations (L/S alleles), while the missense variants in the coding region of *SLC6A4*are not included. This is best illustrated by the *SLC6A4* rs25331 variant, as medication recommendation concordance between different DSTs for patients with MDD was found to be partly dependent on whether a PGx test included this variant.^5^ Modeling combinatorial effects across multiple genes is also challenged by differences in ethnicity and clinical characteristics of the patient cohorts used to validate PGx tests. Regardless, PGx testing can help assess risk of side effects and efficacy related to antidepressant use.^26^

## Haplotype determination

**Fig. 2:**
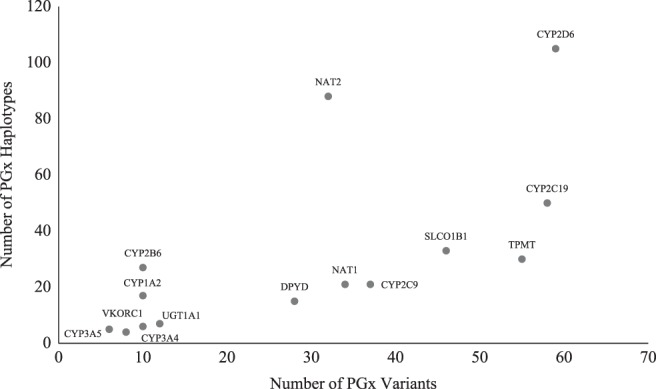
The number of haplotypes versus the number of variants for common PGx genes as curated by PharmGKB. Not all haplotypes follow the “one variant per haplotype” rule, with notable examples being *CYP2D6* and *NAT2*

Variability in drug response can be caused by a single SNP variant or a combination of SNPs (i.e., haplotype) (Fig. 2). The alleles of important drug metabolizing genes, such as *CYP2B6*, *CYP2C9*, *CYP2C19*, and *CYP2D6*, are defined by haplotypes. The more variants measured within a haplotype, the greater the accuracy of allele determination. *CYP2D6* haplotypes are often defined by multiple SNPs. For example, the CYP2D6*64 allele is characterized by 100C>T, as well as 1023C>T and 2850C>T, which are also associated with CYP2D6*17.^27^

Ideally, for haplotype assignment, maternal and paternal sequences are read independently. This distinction between maternal and paternal derived haplotypes is known as phase. Depending on the allele, phase can be important (Supplementary Fig. 1).

### Computational phasing

Short reads and genotyping data are often unable to resolve maternal and paternal haplotype information. Therefore, determining phase requires additional computational interpretation. This process is known as phasing.

**Fig. 3:**
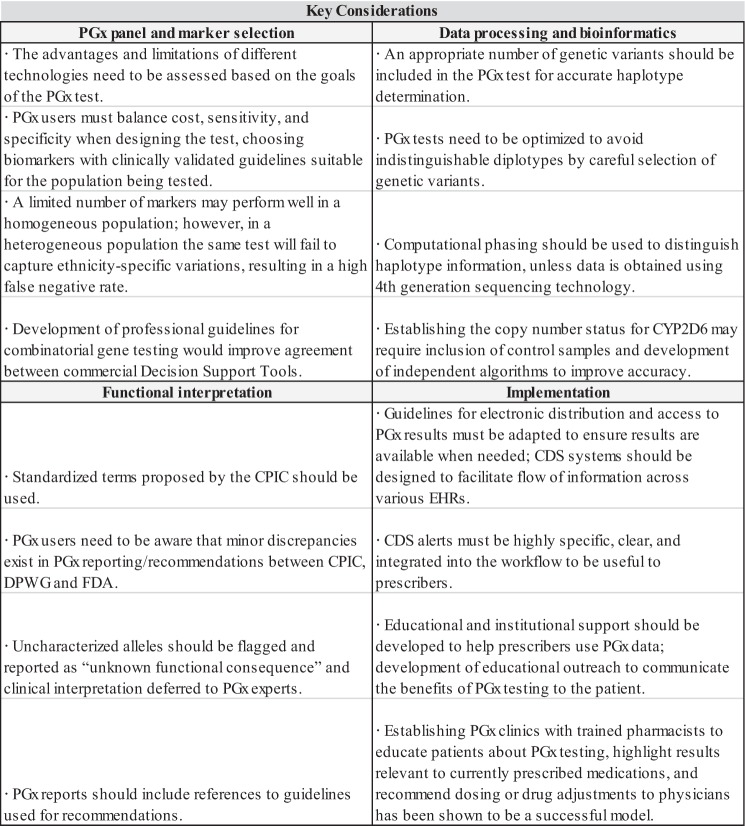
Summary of key considerations underlying four broad areas of pharmacogenomics: PGx panel and marker selection, data processing and bioinformatics, functional interpretation, implementation

Phasing genotyping or sequencing data is achieved computationally through bioinformatics algorithms that rely on statistical inference. A few well-known algorithms have been designed to complete this task, such as FastPHASE,^28^ SHAPE-IT,^29^ BEAGLE,^30^ MACH,^31^ and IMPUTE2.^32^

### Indistinguishable haplotypes

Phasing complex haplotypes can be challenging due to the inability to computationally distinguish between a haplotype pair contained in the corresponding diplotype. For instance, the two alleles *1 and *4 of *CYP2D6* would be indistinguishable as a diplotype from *4M and *10 based on genotyping data and most sequencing data. This could lead to misinterpretation as *1/*4 has an activity score of 1, while *4M/*10 has an activity score of 0.25–0.5.^33^ (The activity score system is used to assign functional status to alleles, i.e., a value of 0.5 corresponds to decreased function). One solution is to design genotyping assays that avoid indistinguishable diplotypes by carefully selecting variants. Commercial PGx assays often do not tend to be optimized in this fashion.

The most complete solution to haplotype-related issues is to utilize technologies that are based on long reads, sufficient to span the distance between markers of interest. Although fourth generation sequencing technologies are available, such as nanopore, they are not currently in wide use due to cost restrictions and error rates.

## Copy number variant (CNV) determination

Copy number variants (CNVs) occur when there are fewer or more than two functional copies of genes. CNVs in pharmacogenes like CYP2D6 impact the activity of the enzyme and as a result, the efficacy and toxicity of their substrates. Since CYP2D6 metabolizes over 25% of currently prescribed drugs, establishing the copy number status and specific alleles duplicated for CYP2D6 is critical for PGx testing.

Various technologies can detect CNVs: high-density SNP genotyping platforms, multiplex RT-PCR, next-generation sequencing. All these technologies, however, have limitations.

Next generation sequencing allows for inspection of ratios between reference and alternative allele reads to deduce allele copy numbers at positions of interest. However, accurate CNV determination typically requires a high depth of coverage which increases cost. Read length and fragment length affect accuracy of CNV determination with paired-end sequencing being more accurate than single-end sequencing. Interpretation of WGS data requires significant bioinformatic resources, while algorithms for CNV determination from whole-exome (WES) data are not fully developed with no current established standard. Fortunately, algorithms are constantly being developed and improved, EXCAVATOR2 and ExCNVSS are two notable examples.

Genotyping assays using the MassARRAY® system (Agena Bioscience, San Diego, CA, USA) or QuantStudio™ (Applied Biosystems™) can detect *CYP2D6* copy number using integrated estimates from other copy number assays and informative polymorphisms between *CYP2D6* and *CYP2D8*. Although assays of smaller batches can sometimes result in inconclusive CNV determination, inclusion of control samples of known *CYP2D6* gene copy status can resolve the issue. Furthermore, development of algorithms independent of the software provided by equipment manufacturers have been found to increase the accuracy of CNV determination for smaller sample batches.

## Functional interpretation; translating PGx results into clinical action

Standardization of variant function and phenotype is a crucial step towards the implementation of PGx. The CPIC has proposed standardized terms to improve the understanding and interpretation of pharmacogenetic test reporting and reduce confusion by maintaining consistent nomenclature.^6^ Functional annotations for pharmacogenes are available at CPIC (https://cpicpgx.org/), PharmGKB (https://www.pharmgkb.org/), DPWG (https://www.pharmgkb.org/page/dpwg), and the Pharmacogene Variation Consortium^34^ (https://www.pharmvar.org/).

### Activity scores assign phenotype; occasional divergence

To facilitate translation of genotype into phenotype, the Activity Score system (AS) developed by Gaedigk et al.^35^ for CYP2D6 is widely accepted. It has been adopted by CPIC for most drug/gene pair recommendations with the activity score determining the functional classification/phenotype to facilitate clinical use. The published consensus terms describing allele functional status and inferred phenotype are listed in Tables 1 and 2.^36^

**Table 1:** Activity score determination for CYP2D6 drug metabolizing enzyme

Allele functional status	Example allele	Allele activity score
Normal function	CYP2D6*1	1
Decreased function	CYP2D6*9	0.5
No function	CYP2D6*4	0
**Phenotype**	**Diplotype combination**	**Diplotype activity score**
Ultra-rapid metabolizer	Two normal function alleles + gene duplication	>2
Normal metabolizer	Two normal function alleles	2
	One normal function + one decreased function alleles	1.5
	One normal function + one loss of function alleles	1
	Two decreased function alleles	1
Intermediate metabolizer	one loss of function + one decreased function alleles	0.5
Poor metabolizer	Two loss of function alleles	0

**Table 2:** Consensus terms to describe three classes of pharmacogenes: drug-metabolizing enzymes, transporters, VKORC1, and high-risk genotypes. Table adapted from Caudle et al^36^

Class	Final term	Functional definition	Genetic definition	Examples
Drug metabolizing enzymes (CYP2C19, CYP2D6, CYP3A5, CYP2C9, TPMT, DPYD, UGT1A1)	Ultrarapid metabolizer	Increased enzyme activity compared to rapid metabolizers	Two increased function alleles, or more than two normal function alleles	CYP2C19*17/*17 CYP2D6*1/*1XN
	Rapid metabolizer	Increased enzyme activity compared to normal metabolizers, but less than ultrarapid metabolizers	Combinations of normal function and increased function alleles	CYP2C19*1/*17
	Normal metabolizer	Fully functional enzyme activity	Combinations of normal function and decreased function alleles	CYP2C19*1/*1
	Intermediate metabolizer	Decreased enzyme activity (activity between normal and poor metabolizer)	Combinations of normal function, decreased function, and/or no function alleles	CYP2C19*1/*2 CYP2C19*2/*17
	Poor metabolizer	Little to no enzyme activity	Combination of no function alleles and/or decreased function alleles	CYP2C19*2/*2
Transporters (SLCO1B1)	Increased function	Increased transporter function compared to normal function	One or more increased function alleles	SLCO1B1*1/*14
	Normal function	Fully functional transporter function	Combinations of normal function and/or decreased function alleles	SLCO1B1*1/*1
	Decreased function	Decreased transporter function (function between normal and poor function)	Combinations of normal function, decreased function, and/or no function alleles	SLCO1B1*1/*5
	Poor function	Little to no transporter function	Combination of no function alleles and/or decreased function alleles	SLCO1B1*5/*5
VKORC1*	G3673A (rs9923231)	Risk allele (A) believed to be the causative SNP for the low-dose warfarin phenotype	GG, GA, AA	VKORC1*2
	C6484T (rs9934438)	Risk allele (T) used as marker for the low-dose warfarin phenotype; in near perfect LD with G3673A	CC, CT, TT	VKORC1*2
	G9041A (rs7294)	Presence of A allele associated with the high-dose warfarin phenotype	GG, GA, AA	VKORC1*3 VKORC1*4
High-risk genotype status (HLA-B)	Positive	Detection of high-risk allele	Homozygous or heterozygous for high-risk allele	HLA-B*15:02
	Negative	High-risk allele not detected	No copies of high-risk allele	

* CPIC does not have consensus terms for VKORC1

However, the classification of cytochrome enzymes into just three or four categories (poor metabolizer, intermediate metabolizer, normal/extensive metabolizer, and ultrarapid metabolizer) can pose challenges, especially in the translation of highly complex CYP2D6 genotype data into a patient’s phenotype to guide drug therapy.^35^ Some genotypes may fall somewhere in-between the categories mentioned above, leading to discrepancies in functional interpretation by different groups. CPIC and DPWG differ in their translation of genotype to phenotype for some alleles in CYP2D6, CYP2C9, CYP2C19, and DPYD.^14^ For example, DPWG assigns CYP2D6*1/*5 into the intermediate metabolizer category, while CPIC classifies CYP2D6*1/*5 as normal metabolizer.

Creating additional functional categories can potentially improve resolution of PGx drug dosing. In July 2016, CPIC introduced the additional phenotype, ‘rapid metabolizer’ to distinguish between individuals with a CYP2C19 *1/*17 (rapid metabolizer) and CYP2C19 *17/*17 (ultrarapid metabolizer). This new phenotype provides therapeutic recommendations on the gene-drug interaction of CYP2C19 and voriconazole.^37^ Such expansion is justified if new categories help delineate differences or altered pharmacokinetics of a probe drug.

Exceptionally rare coding variants that have not been functionally characterized can also pose a challenge in functional interpretation. While currently there is no consensus on how to handle uncharacterized alleles, these should be flagged and reported as ‘unknown functional consequence’ to alert health-care providers and defer clinical interpretation to PGx experts.

### Guidelines; occasional discordance in recommendations

PharmGKB annotates PGx-based drug dosing guidelines published by CPIC, DPWG, CPNDS and other professional societies, while the FDA provides a list of drugs that contain information on PGx biomarkers in drug labels.

A recent publication comparing therapeutic recommendations for well-known gene-drug pair by CPIC and DPWG found substantial agreement between recommendations by the two consortia.^14^ However, differences in therapeutic recommendations were noted for one or more aberrant phenotypes in 13 gene-drug pairs (see Bank et al.^14^ for a detailed list of discordant guidelines). Some differences were due to variance in clinical practices between countries. Others were due to the ‘time effect’, literature searches being performed at different time points by the two consortia while new articles are published continuously, underscoring the need to continually update existing recommendations.

Guidelines can differ slightly between CPIC, DPWG, and the US FDA’s ‘black box warning’. Guidelines for clopidogrel, an antiplatelet agent, by CPIC and the DPWG recommend alternative antiplatelet therapy for intermediate and poor metabolizers of CYP2C19. In contrast, the FDA’s ‘black box warning’ on clopidogrel, the strictest warning in labeling of prescription drugs designed to call attention to serious or life-threatening risks, states a danger for lower effectiveness only in CYP2C19 poor metabolizers.

Prescribers can be made aware of PGx-related information, even if specific dosing recommendations are not available. The FDA labels biomarker information for carisoprodol, a muscle relaxant, as ‘Actionable PGx’, as patients with reduced CYP2C19 activity may have a fourfold increase in exposure to carisoprodol with a concomitant 50% decreased exposure to meprobamate (a metabolite of carisoprodol) compared to normal CYP2C19 metabolizers. Prescribers can use such drug label information, if compiled into a single PGx report, to make informed decisions.

PGx reports should include references to the sources used for guidelines, such that prescribers can weigh the strength of evidence available.

## Clinical implementation

To implement PGx into clinical practice, timely sharing of information and educational support must be made available to health-care providers. PGx implementation requires digital storage and secure, prompt accessibility of information to authorized users, often with PGx data embedded as part of an electronic health record (EHR) system. Early adopters of PGx programs developed their own implementation strategies and clinical decision support (CDS) systems. CDS systems are used to provide patient-specific PGx recommendations, and can be integrated into EHR. However, usability evaluations indicate that multi-institutional efforts are warranted to develop relevant guidelines.^38^

Worldwide, many PGx groups are sharing resources to further develop implementation guidelines. The European Pharmacogenetics Implementation Consortium (http://www.eu-pic.net/) has undertaken efforts to facilitate PGx implementation in clinical practice, as well as the Royal Dutch Association for the Advancement of Pharmacy. The National Institutes of Health’s Pharmacogenomics Research Network, and associated eMERGE and IGNITE networks, have initiated several PGx implementation programs across different US sites and are studying outcomes to develop a consensus strategy. St. Jude Children’s Research Hospital (PG4KDS protocol) and the University of Chicago (The 1200 Patients Project) are developing model systems for the clinical implementation of preemptive PGx.

Many commercially available PGx-based DSTs make test results available in a portable document format (pdf). The secure transfer of PGx test results and structured patient-specific dosing recommendations to prescribers for both current and future use is needed for the lifetime value of PGx tests to be realized. Some health-care providers have developed PGx alerts for EMRs, while others use web-based applications.

### Strategies for PGx implementation

A consensus strategy to aid PGx implementation into clinical care has emerged from the National Institutes of Health-funded IGNITE (Implementing GeNomics In pracTicE) network.^39,40^ Three different IGNITE institutions (Indiana University, University of Florida, and Vanderbilt University) implemented PGx in real-world clinical settings using data warehousing techniques to adapt and tailor innovations to various contexts, with sites extracting data from multiple sources to integrate clinical records across organizations into a central repository (Table 3).

Indiana University conducted PGx testing for 24 widely used drugs in a hospital setting and evaluated cost reductions over 1 year. The University of Florida partnered with primary care professionals across the state to implement PGx as part of routine patient care. Vanderbilt University Medical Center developed CDS within adopter sites to select and genotype prospective patients.

**Table 3:** Comparison of data warehousing and CDS for PGx implementation projects^40–42^

Site/project	Data storage and security	CDS
Indiana University—INGenious: INdiana Genomics Implementation: an Opportunity for the UnderServed	Data in secured database and Eskanzi EHR	• Automatic alerts • Links to guidelines and supporting evidence for patients with pharmacogenomic results
University of Florida—UF Health Personalized Medicine Program	Clinical data in EHR; secure facilities	• Alert-based informed message that integrates EHR and allele data • Includes link to patient education materials
Vanderbilt University—Integrated, Individualized and Intelligent Prescribing (I3P) Network	Data stored on individual site servers; Veterans Affairs site data on FISMA compliant server	• Passive and active alerts • Includes interpretative recommendations • Link to external information sources (e.g., MyCancerGenome.org)
St. Jude Children’s Research Hospital—PG4KDS protocol	Data posted to EHR through in-house custom web-based applications, DMET Tracker and Consult Builder	• Active alerts presented for high-risk drugs with recommendations to guide prescribing
University of Chicago—The 1200 Patients Project	Clinical data in protected-access web-portal, the genomic prescribing system (GPS)	• Patient-specific drug interpretation as summary providers can read in <30 s, dynamic feature allows system use in real time as new treatments considered

From their experience, the following consensus strategy emerged for PGx implementation: (i) integrate genomic results into EHRs, and provide CDS; (ii) educate prescribers to effectively use PGx information; and (iii) engage patients.

With PG4KDS, St. Jude’s successfully implemented preemptive PGx in over 1000 patients. Key elements of success included a process to manage return of results and incidental findings, extensive use of informatics, development of EHR and CDS, and broad clinician education efforts.^41^

The University of Chicago’s 1200 patient project model relied on a point-of-care informatics support, the genomic prescribing system (GPS) to bridge: (i) information dissemination and provider education; (ii) instantaneous availability of results; and (iii) clinical interpretation and guidance. To demonstrate the acceptance and feasibility of PGx use in busy clinical settings, ‘early-adopter’ physicians were recruited for the 1200 patient project. At each patient visit, providers were monitored on their access of the GPS to query PGx information during treatment decision-making. By studying early-adopter provider-patient pairs incorporating a broad range of PGx information, the project hopes to gain important insights into the PGx implementation processes.^42^

Despite significant progress, reported challenges still need to be overcome. Currently, there are no standard methods for creating alerts about actionable variants and each site in the IGNITE network created their own CDS rules.^39^

### CDS: challenges and solutions

CDS systems can be designed as separate programs, web services, or mobile applications. CDS systems that are tailored for use within the local EHR are often restricted to the respective local health IT infrastructure. Although some genomic data-sharing standards have been developed, commercial EHR platforms have been slow to incorporate the standards or to facilitate the flow of structured information across different systems.

A CDS system developed by The Ubiquitous Pharmacogenomics (U-PGx) Consortium presents a solution for mobilizing PGx data that also engages patients in their own health care. The U-PGx Consortium, funded by the European Union, evaluated tools to integrate PGx test results across health-care institutions in seven European countries.^43^ A flexible mobile-based CDS system, entitled the Medication Safety Code (MSC), was evaluated among physicians and pharmacists. The MSC system stores PGx data in two-dimensional quick response codes to be interpreted by smartphones and other devices. The MSC system, which is provided as a personalized pocket card carried by the patient, was successfully used to alert physicians and pharmacists to PGx recommendations.^44^

The International (IFCC) Pharmacogenetics Expertcenter at the Department of Clinical Chemistry, Erasmus MC, Rotterdam, has used personalized pocket cards since 2013 to transfer genotype information to the physician and pharmacist: making the patient the carrier of their information. Although this approach is limited in the amount of information that can be mobilized, its use in the Netherlands has been successful since dosing information per genotype is already available at every pharmacist through the DPWG guidelines that are incorporated in a national network.

### Educational support for primary care prescribers

Primary care prescribers are, in general, unfamiliar with PGx data. Educational support for prescribers to feel comfortable using PGx data to make clinical decisions is vital for the success of any PGx program. In two surveys conducted in the US, physicians had reported near-universal acceptance of the concept of PGx, but had rarely been educated on the topic and felt unprepared for ordering and using test results.^45,46^ The response of primary care physicians to PGx CDS alerts indicated that many did not find them useful.^47^ More than 50% found the alerts confusing, and had difficulty in locating additional information. Hence only 30% of the prescribers that received a CDS alert changed their prescription to an alternative medication. Surveys indicated that 45% of primary care prescribers were unsure about the use of PGx CDS in the future.

On the contrary, primary care prescribers in institutional PGx programs felt adequately supported to use the results in their clinical practice.^48^ These prescribers had attended educational seminars, received informational brochures, and had direct communications with PGx program leaders. A total 99% of prescribers agreed that PGx variants influence patient response to drug therapy. However, they could not agree on how to assign clinical responsibility for actionable results, indicating prescribers do not feel comfortable with genetic information not directly related to their specialty.

Pharmacists can be assigned clinical responsibility for actionable PGx results, and can play a key role in helping primary care prescribers deliver and interpret PGx testing. A pharmacist-led surveillance team reviewed electronic records of CYP2C19 variant status for patients who were prescribed clopidogrel following a coronary stent.^49^ Pharmacists directly messaged attending physicians using a system built into the EHR. Cardiologists receiving direct notification of CYP2C19 status for patients with a variant affecting clopidogrel metabolism suggested alternative medications. Over a 12-month period, 58% of poor metabolizers and 33% of intermediate metabolizers received alternatives to clopidogrel, with CYP2C19 variant status being the most influential factor impacting prescribing decisions.

### Patient engagement

Personalized medicine is essentially individualized and therefore an opportunity to engage patients in their own health care. To realize the anticipated lifetime benefits of PGx, the results will need to be shared with health-care providers belonging to a patients’ circle of care. Although PGx has gained considerable recognition, physicians are still reluctant to adopt PGx into routine practice. Increased availability of direct-to-consumer PGx testing has led to patients taking initiative to utilize the benefits of PGx. Pre-emptive commercial PGx tests can provide value for patients, covering multiple commonly prescribed medications in anticipation of future prescription events. However, the ‘burden of disclosure’ often becomes the patients’ responsibility.^50^ Strategies to communicate the beneficial aspects of genomic medicine need to be developed to fully engage patients as potential drivers of PGx utilization.

## Conclusion

The expansion of available genomic data has led to a rapid increase in the number of PGx variants identified. Efforts to assign function and determine dosing recommendations for these variants remains an ongoing effort. Despite significant progress in standardizing different aspects of PGx testing, panels vary in the pharmacogenomic variants included, affecting test specificity and sensitivity, leading to a confusing marketplace with many apparently similar panels offering different value. A lack of understanding of the underlying technology of a PGx test often results in prescribers and other potential users of PGx viewing the validity of PGx testing with mistrust, rather than as a tool that leads to better drug outcomes and increases the quality of patient care. Greater familiarity with key technological aspects can help potential users gain acceptance of PGx, and facilitate discernment of which PGx tests better suits their needs. PGx is not an absolute science, but provides opportunities for informed decision-making; and PGx results can be used along with other clinical criteria for prescribing decisions. This approach is exemplified by warfarin-dosing algorithms that use both genetic and non-genetic factors to individualize warfarin doses.^51^ However, more work needs to be done to overcome barriers to implementation before PGx testing can become standard-of-care like any other biochemical test offered by health-care providers (Fig. 3). Efforts continue to bridge the gap between the science of PGx and real-world application, as wide-spread implementation moves closer to realization.

## Electronic supplementary material

Supplementary Material

## Data Availability

Data sharing not applicable to this article as no datasets were generated or analyzed.

## Electronic supplementary material

**Supplementary information** accompanies the paper on the *npj Genomic Medicine* website (10.1038/s41525-018-0065-4).
