# Supplementary material for: User considerations in assessing pharmacogenomic tests and their clinical support tools
Source: NPJ Genom Med. 2018 Sep 11;3:26. doi: 10.1038/s41525-018-0065-4 (PMC6133969; doi:10.1038/s41525-018-0065-4)
Supplement: Supplementary file 1 — Supplementary Material [file 41525_2018_65_MOESM1_ESM.docx]

Supplemental Table 1: Comparison of different PGx panels offered by commercial vendors.

| **gene** | **rs number** | **vendor1** | **vendor2** | **vendor3** | **vendor4** | **vendor5*** | **vendor6*** | **vendor7*** |
| --- | --- | --- | --- | --- | --- | --- | --- | --- |
| ABCB1 | rs1045642 |  | ✓ | ✓ |  | ✓ |  |  |
|  | rs1128503 |  | ✓ |  |  |  |  |  |
|  | rs2032582 |  | ✓ | ✓ |  |  |  |  |
|  | rs3213619 |  | ✓ |  |  |  |  |  |
| ABCG2 |  |  |  | ✓ |  |  |  |  |
| ADD1 |  |  |  | ✓ |  |  |  |  |
| ADRB2 | rs1042713 |  |  | ✓ |  |  |  | ✓ |
| ANK3 |  |  |  |  |  |  |  | ✓ |
| ANKK1 |  |  |  | ✓ |  |  |  |  |
| APOE | rs7412 |  |  |  |  | ✓ |  |  |
|  | rs429358 |  |  |  |  | ✓ |  |  |
| ATIC |  |  |  | ✓ |  |  |  |  |
| BDNF |  |  |  |  |  |  |  | ✓ |
| C11orf65 |  |  |  | ✓ |  |  |  |  |
| CACNA1C |  |  |  |  |  |  |  | ✓ |
| COMT | rs4680 | ✓ |  | ✓ |  |  |  | ✓ |
| CYP1A2 | rs2069514 | ✓ |  |  |  | ✓ | ✓ | ✓ |
|  | rs2069526 | ✓ | ✓ |  |  |  | ✓ | ✓ |
|  | rs12720461 | ✓ | ✓ |  |  | ✓ | ✓ | ✓ |
|  | rs35694136 | ✓ |  |  |  |  |  |  |
|  | rs762551 | ✓ | ✓ |  | ✓ | ✓ | ✓ | ✓ |
|  | rs28399424 |  | ✓ |  |  |  | ✓ |  |
|  | rs56107638 |  | ✓ |  |  | ✓ | ✓ |  |
|  | rs72547513 |  |  |  |  | ✓ |  | ✓ |
|  | rs72547511 |  | ✓ |  |  |  | ✓ | ✓ |
|  | rs72547517 |  | ✓ |  |  |  | ✓ | ✓ |
| CYP2A6 | rs28399453 |  |  |  |  |  |  |  |
|  | rs150298687 |  |  |  |  |  |  |  |
|  | rs57837628 |  |  |  |  |  |  |  |
|  | rs56113850 |  |  |  |  |  |  |  |
|  | rs7260629 |  |  |  |  |  |  |  |
| CYP2B6 | rs3211371 | ✓ | ✓ |  |  |  |  | ✓ |
|  | rs3745274 | ✓ | ✓ |  |  | ✓ | ✓ | ✓ |
|  | rs2279343 | ✓ |  |  |  |  |  | ✓ |
|  | rs28399499 | ✓ | ✓ |  |  | ✓ |  |  |
|  | rs12721655 |  | ✓ |  |  |  | ✓ | ✓ |
|  | rs34223104 |  | ✓ |  |  |  |  |  |
|  | rs8192709 |  | ✓ |  |  |  |  |  |
| CYP2C cluster | rs12248560 | ✓ | ✓ |  |  | ✓ |  |  |
| CYP2C19 | rs28399504 | ✓ | ✓ | Gene tested but markers are not disclosed | ✓ |  | ✓ | ✓ |
|  | rs4986893 | ✓ | ✓ |  |  |  | ✓ | ✓ |
|  | rs6413438 | ✓ | ✓ |  | ✓ |  | ✓ | ✓ |
|  | rs4244285 | ✓ | ✓ |  |  | ✓ | ✓ | ✓ |
|  | rs41291556 | ✓ | ✓ |  | ✓ |  | ✓ | ✓ |
|  | rs72552267 |  | ✓ |  |  |  | ✓ | ✓ |
|  | rs12777823 |  | ✓ |  |  |  | ✓ | ✓ |
| CYP2C8 | rs1058930 |  | ✓ |  |  |  |  |  |
|  | [rs11572080](https://www.pharmgkb.org/rsid/rs267606617) |  | ✓ |  |  |  |  |  |
|  | [rs11572103](https://www.pharmgkb.org/variant/PA166159181) |  | ✓ |  |  |  |  |  |
| CYP2C9 | rs28371685 | ✓ | ✓ | Gene tested but markers are not disclosed |  |  |  | ✓ |
|  | rs1057910 | ✓ | ✓ |  | ✓ | ✓ | ✓ | ✓ |
|  | rs56165452 | ✓ |  |  |  | ✓ | ✓ | ✓ |
|  | rs28371686 | ✓ |  |  |  | ✓ | ✓ | ✓ |
|  | rs1057911 | ✓ |  |  |  |  |  | ✓ |
|  | rs1799853 | ✓ | ✓ |  | ✓ | ✓ | ✓ | ✓ |
|  | rs7900194 | ✓ |  |  |  |  |  | ✓ |
|  | rs9332131 | ✓ |  |  |  | ✓ | ✓ | ✓ |
|  | rs2256871 |  | ✓ |  |  | ✓ |  |  |
|  | rs72558190 |  |  |  |  | ✓ |  |  |
|  | rs72558187 |  |  |  |  | ✓ |  |  |
|  | rs9332239 |  | ✓ |  |  | ✓ |  |  |
| CYP2D6** | rs1080985 | ✓ |  | Gene tested but markers are not disclosed |  |  | ✓ | ✓ |
|  | rs1065852 | ✓ |  |  |  | ✓ | ✓ | ✓ |
|  | rs59421388 | ✓ | ✓ |  | ✓ | ✓ | ✓ | ✓ |
|  | rs72549346 | ✓ | ✓ |  | ✓ | ✓ | ✓ | ✓ |
|  | rs5030862 | ✓ |  |  |  | ✓ | ✓ | ✓ |
|  | rs267608319 | ✓ |  |  |  |  | ✓ | ✓ |
|  | rs774671100 | ✓ |  |  |  |  | ✓ | ✓ |
|  | rs765776661 | ✓ |  |  |  |  | ✓ | ✓ |
|  | rs1135840 | ✓ |  |  |  |  | ✓ | ✓ |
|  | rs201377835 | ✓ |  |  | ✓ | ✓ | ✓ | ✓ |
|  | rs769258 | ✓ |  |  |  |  | ✓ | ✓ |
|  | rs28371706 | ✓ |  |  |  | ✓ | ✓ | ✓ |
|  | rs5030655 | ✓ | ✓ |  | ✓ | ✓ | ✓ | ✓ |
|  | rs5030865 | ✓ | ✓ |  | ✓ | ✓ | ✓ | ✓ |
|  | rs3892097 | ✓ |  |  |  |  | ✓ | ✓ |
|  | rs72549354 | ✓ |  |  |  |  | ✓ | ✓ |
|  | rs72549353 | ✓ |  |  |  |  | ✓ | ✓ |
|  | rs35742686 | ✓ |  |  |  |  | ✓ | ✓ |
|  | rs5030656 | ✓ | ✓ |  | ✓ | ✓ | ✓ | ✓ |
|  | rs16947 | ✓ |  |  | ✓ | ✓ | ✓ | ✓ |
|  | rs5030867 | ✓ |  |  | ✓ | ✓ | ✓ | ✓ |
|  | rs72549357 |  |  |  |  | ✓ |  |  |
|  | rs79292917 | ✓ | ✓ |  | ✓ | ✓ | ✓ | ✓ |
|  | rs28371725 | ✓ |  |  |  |  | ✓ | ✓ |
| CYP3A4 | rs2740574 | ✓ |  |  |  |  |  |  |
|  | rs35599367 | ✓ | ✓ |  | ✓ | ✓ | ✓ | ✓ |
|  | rs12721627 | ✓ | ✓ |  |  |  |  |  |
|  | rs12721629 |  | ✓ |  |  |  |  |  |
|  | rs28371759 |  | ✓ |  |  |  |  |  |
|  | rs4646438 |  | ✓ |  |  |  |  |  |
|  | rs4986910 |  | ✓ |  |  |  |  |  |
|  | rs4986913 |  | ✓ |  |  |  |  |  |
|  | rs55785340 |  | ✓ |  |  | ✓ |  |  |
|  | rs4987161 |  | ✓ |  |  | ✓ |  |  |
|  | rs4986907 |  |  |  |  |  | ✓ |  |
|  | rs4986909 |  |  |  |  |  | ✓ |  |
|  | rs67784355 |  | ✓ |  |  |  |  |  |
| CYP3A5 | rs41303343 |  | ✓ |  |  | ✓ |  | Gene tested but markers are not disclosed |
|  | rs776746 | ✓ | ✓ |  | ✓ | ✓ |  |  |
|  | rs10264272 | ✓ | ✓ |  |  | ✓ |  |  |
|  | rs28365083 | ✓ | ✓ |  |  | ✓ |  |  |
|  | rs28383468 |  | ✓ |  |  |  |  |  |
| CYP4F2 | rs2108622 | ✓ |  | ✓ |  |  |  |  |
| DPP6 |  |  |  | ✓ |  |  |  |  |
| DPYD | rs55886062 | ✓ |  |  |  |  |  |  |
|  | rs3918290 | ✓ | ✓ |  |  |  |  |  |
|  | rs67376798 | ✓ | ✓ |  |  |  |  |  |
|  | rs1801158 |  | ✓ |  |  |  |  |  |
|  | rs1801159 |  | ✓ |  |  |  |  |  |
|  | rs1801160 |  | ✓ |  |  |  |  |  |
|  | rs1801265 |  | ✓ |  |  |  |  |  |
| DRD2 | rs1799978 | ✓ |  | Markers not disclosed |  |  |  | Markers not disclosed |
|  | rs1800497 |  |  |  |  | ✓ |  |  |
|  | rs1076560 |  |  |  |  |  |  |  |
| F2 | rs1799963 |  |  | ✓ |  | ✓ |  |  |
| F5 | rs6025 | ✓ | ✓ | ✓ |  | ✓ |  |  |
| FDPS |  |  |  | ✓ |  |  |  |  |
| FKBP5 |  |  |  | ✓ |  |  |  |  |
| G6PD |  |  |  | ✓ |  |  |  |  |
| GLP1R | rs1042044 |  |  |  |  | ✓ |  |  |
|  | rs6923761 |  |  |  |  | ✓ |  |  |
|  | rs2300615 |  |  |  |  | ✓ |  |  |
| GNB3 |  |  |  | ✓ |  |  |  |  |
| GRIK1 |  |  |  |  |  |  |  | ✓ |
| GRIK4 | rs1954787 | ✓ |  | ✓ |  |  |  | ✓ |
| HLA-A *31:01 | rs1061235 | ✓ |  | ✓ |  |  | ✓ |  |
| HLA-B | HLA00386 | ✓ |  |  |  |  | ✓ |  |
|  | rs144012689 | ✓ |  |  |  |  | ✓ |  |
| HLA-B:1502 |  | ✓ |  |  |  |  | ✓ |  |
| HTR1A | rs6295 | ✓ |  | ✓ |  |  |  |  |
| HTR2A | rs7997012 | ✓ |  |  |  |  | ✓ |  |
| HTR2C | rs3813929 | ✓ |  | ✓ |  |  |  | Markers not disclosed |
|  | rs1414334 |  |  | ✓ |  |  |  |  |
| IFNL3/4 | rs12979860 | ✓ | ✓ |  |  |  |  |  |
| MC4R | [rs489693](https://www.pharmgkb.org/rsid/rs489693) |  |  | ✓ |  |  |  | ✓ |
|  | rs17782313 |  |  |  |  |  |  |  |
| MTHFR | rs1801131 | Optional |  |  |  | ✓ |  | ✓ |
|  | rs1801133 | Optional |  |  |  | ✓ |  | ✓ |
| NUDT15 | rs116855232 | ✓ |  | ✓ |  |  |  |  |
| OPRM1 | rs1799971 | ✓ | ✓ | ✓ |  | ✓ |  | ✓ |
|  | rs34427887 |  | ✓ |  |  |  |  |  |
|  | rs62638690 |  | ✓ |  |  |  |  |  |
| PRKCA |  |  |  | ✓ |  |  |  |  |
| RAR | rs2229774 |  |  |  |  |  |  |  |
| RNR1 | rs267606617 |  |  | ✓ |  |  |  |  |
|  | rs267606619 |  |  | ✓ |  |  |  |  |
| SLC28A3 |  |  |  |  |  |  |  |  |
| SLC47A2 |  |  |  | ✓ |  |  |  |  |
| SLC6A4 | rs774676466 | ✓ |  |  |  |  |  |  |
|  | rs25531 | ✓ |  |  |  |  | ✓ | ✓ |
| SLCO1B1 | rs4149015 | ✓ | ✓ | Gene tested but markers not disclosed | ✓ | ✓ |  |  |
|  | rs2306283 | ✓ | ✓ |  |  |  |  |  |
|  | rs4149056 | ✓ | ✓ |  |  |  |  |  |
|  | rs59502379 |  | ✓ |  |  |  |  |  |
| TPMT | rs1800462 | ✓ | ✓ |  |  |  |  |  |
|  | rs1800460 | ✓ | ✓ |  |  |  |  |  |
|  | rs1800584 | ✓ |  |  |  |  |  |  |
|  | rs1142345 | ✓ |  |  |  |  |  |  |
|  | rs56161402 |  | ✓ |  |  |  |  |  |
| UGT1A1 | rs4148323 | ✓ | ✓ |  |  |  |  |  |
|  | rs1976391 | ✓ |  |  |  |  |  |  |
|  | rs887829 |  | ✓ |  |  |  |  |  |
|  | rs35350960 |  | ✓ |  |  |  |  |  |
|  | rs4124874 |  | ✓ |  |  |  |  |  |
| UGT1A4 | rs2011425 |  |  |  |  |  |  | ✓ |
|  | rs3732218 |  |  |  |  |  |  | ✓ |
|  | rs3732219 |  |  |  |  |  |  | ✓ |
| UGT1A6 | [rs1105879](https://www.pharmgkb.org/variant/PA166155623) |  |  |  |  |  |  | ✓ |
|  | rs17863783 |  |  |  |  |  |  | ✓ |
|  | [rs2070959](https://www.pharmgkb.org/variant/PA166155665) |  |  |  |  |  |  | ✓ |
|  | rs7853758 |  |  |  |  |  |  | ✓ |
|  | [rs6759892](https://www.pharmgkb.org/variant/PA166155760) |  |  |  |  |  |  | ✓ |
|  | [rs4261716](https://www.pharmgkb.org/variant/PA166155725) |  |  |  |  |  |  | ✓ |
| UGT2B15 | rs1902023 |  |  |  |  |  |  | ✓ |
| VKORC1 | rs9923231 | ✓ | ✓ | ✓ | ✓ | ✓ |  |  |
|  | rs7200749 | ✓ |  |  |  |  |  |  |
|  | rs7294 |  |  | ✓ |  |  |  |  |

*Vendor6 and Vendor7 are psychology only PGx panels. Vendor5 has depression and psychiatry panels available.

**CYP2D6 requires CNV analysis. Without CNV analysis for CYP2D6, some theoretically detected haplotypes are conflicting (and thus ability to report correct diplotypes).


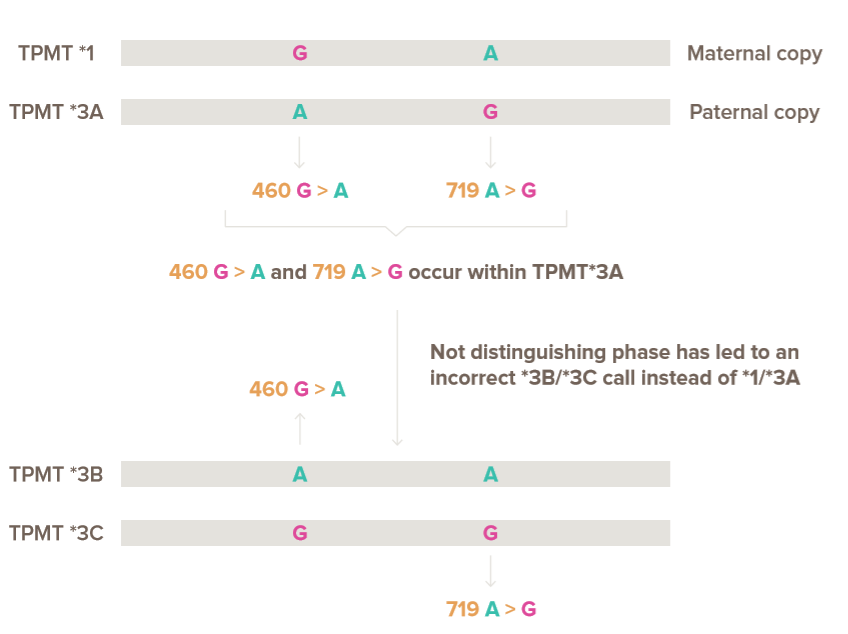
Supplemental Figure 1: Mischaracterization of TPMT alleles resulting from improperly distinguished phase information. Two low enzyme activity alleles, *TPMT*3B* and *TPMT*3C*, are both characterized by SNPs occurring within the *TPMT*3A* allele. In most laboratories, *3B/*3C is now called as *1/*3A due to the very low occurrence of the *3B allele, making the combination *1/*3A 15,000-20,000 times more likely than *3B/*3C. However, such a huge difference in likelihood does not always occur. As more SNPs are determined per patient, the aspect of phasing becomes increasingly important, and allele determination can no longer be left to chance calculations.
